# *In Vitro* Model for Hepatotoxicity Studies Based on Primary Human Hepatocyte Cultivation in a Perfused 3D Bioreactor System

**DOI:** 10.3390/ijms17040584

**Published:** 2016-04-16

**Authors:** Fanny Knöspel, Frank Jacobs, Nora Freyer, Georg Damm, An De Bondt, Ilse van den Wyngaert, Jan Snoeys, Mario Monshouwer, Marco Richter, Nadja Strahl, Daniel Seehofer, Katrin Zeilinger

**Affiliations:** 1 Bioreactor Group, Berlin-Brandenburg Center for Regenerative Therapies (BCRT), Charité-Universitätsmedizin Berlin, Campus Virchow-Klinikum, Berlin 13353, Germany; fanny.knoespel@charite.de (F.K.); nora.freyer@charite.de (N.F.); marco.richter@charite.de (M.R.); nadja.strahl@charite.de (N.S.); 2 Janssen Research & Development, Beerse 2340, Belgium; FJACOBS1@its.jnj.com (F.J.); ADBONDT@its.jnj.com (A.D.B.); IVDWYNGA@its.jnj.com (I.W.); JSNOEYS@its.jnj.com (J.S.); MMONSHOU@ITS.JNJ.COM (M.M.); 3 Department for General, Visceral and Transplantation Surgery, Charité-Universitätsmedizin Berlin, Campus Virchow-Klinikum, Berlin 13353, Germany; georg.damm@charite.de (G.D.); daniel.seehofer@charite.de (D.S.)

**Keywords:** primary human hepatocytes, *in vitro* hepatotoxicity model, three-dimensional (3D) bioreactor, diclofenac

## Abstract

Accurate prediction of the potential hepatotoxic nature of new pharmaceuticals remains highly challenging. Therefore, novel *in vitro* models with improved external validity are needed to investigate hepatic metabolism and timely identify any toxicity of drugs in humans. In this study, we examined the effects of diclofenac, as a model substance with a known risk of hepatotoxicity *in vivo*, in a dynamic multi-compartment bioreactor using primary human liver cells. Biotransformation pathways of the drug and possible effects on metabolic activities, morphology and cell transcriptome were evaluated. Formation rates of diclofenac metabolites were relatively stable over the application period of seven days in bioreactors exposed to 300 µM diclofenac (300 µM bioreactors (300 µM BR)), while in bioreactors exposed to 1000 µM diclofenac (1000 µM BR) metabolite concentrations declined drastically. The biochemical data showed a significant decrease in lactate production and for the higher dose a significant increase in ammonia secretion, indicating a dose-dependent effect of diclofenac application. The microarray analyses performed revealed a stable hepatic phenotype of the cells over time and the observed transcriptional changes were in line with functional readouts of the system. In conclusion, the data highlight the suitability of the bioreactor technology for studying the hepatotoxicity of drugs *in vitro*.

## 1. Introduction

To date, there is an unmet need towards innovative *in vitro* liver models enabling clinical translation of laboratory research. Each new drug has to undergo extensive preclinical testing before entering clinical trials, and on average only one out of nine developed compounds receives approval by the regulatory authorities [1]. Animal models and *in vitro* models are both currently used to investigate pharmacodynamics and pharmacokinetics of new drugs. A major limitation of animal models is the insufficient prediction of human drug effects due to species-dependent differences in hepatic metabolism and different sensitivity to toxic effects [2]. *In vitro* culture models provide an option to address this limitation by using human liver cells. Human hepatoma cell lines were suggested as a potential human cell source for *in vitro* studies due to their high proliferation capacity and good availability [3,4], but aberrations in specific metabolic enzymes can alter bioactivation processes in pharmacological studies. The generation of hepatocytes from human stem cells, such as embryonic or induced pluripotent stem cells, holds large potential for future applications but currently lacks robust protocols for generating hepatocyte-like cells with a sufficiently mature hepatic phenotype [5]. Thus, primary human hepatocytes (pHH) are currently deemed as the gold standard to provide human-predictive data on hepatic metabolism and toxicity of new drugs [6].

**Figure 1:**
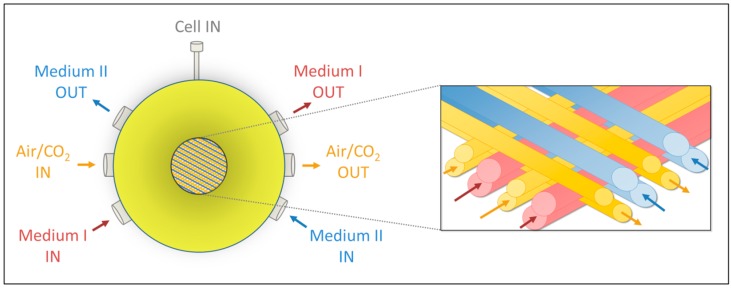
Schematic illustration of the three-dimensional (3D) multi-compartment hollow-fiber bioreactor technology. The bioreactor consists of three independent bundles of hollow fiber membranes interwoven in four layers. Each layer is composed of alternately arranged medium and gas capillaries. The two medium-carrying bundles (**red** and **blue**) are perfused in a counter-current way to enhance mass exchange. The third bundle (**orange**) serves for gas supply of the cells cultured between the capillaries.

The commonly used two-dimensional (2D) cultures of pHH are characterized by a lack of cell–cell contacts and paracrine signaling resulting in a rapid loss of metabolic functions [7,8]. To overcome the limitations of classical 2D culture systems, various 2D co-culture and 3D culture systems for cultivation of pHH were developed to improve the long-term maintenance and functionality of the cells *in vitro* [9,10,11]. Our approach is based on a 3D multi-compartment hollow-fiber bioreactor technology (Figure 1) for high-density perfusion culture of pHH in co-culture with non-parenchymal liver cells [12]. Tissue-like cell reorganization and stable cell maintenance over at least two weeks were observed using different scales of the technology [13]. In addition, stable preservation of cytochrome P450 (CYP) dependent enzyme activities under serum-free conditions was shown, using a miniaturized bioreactor variant [14,15].

**Figure 2:**
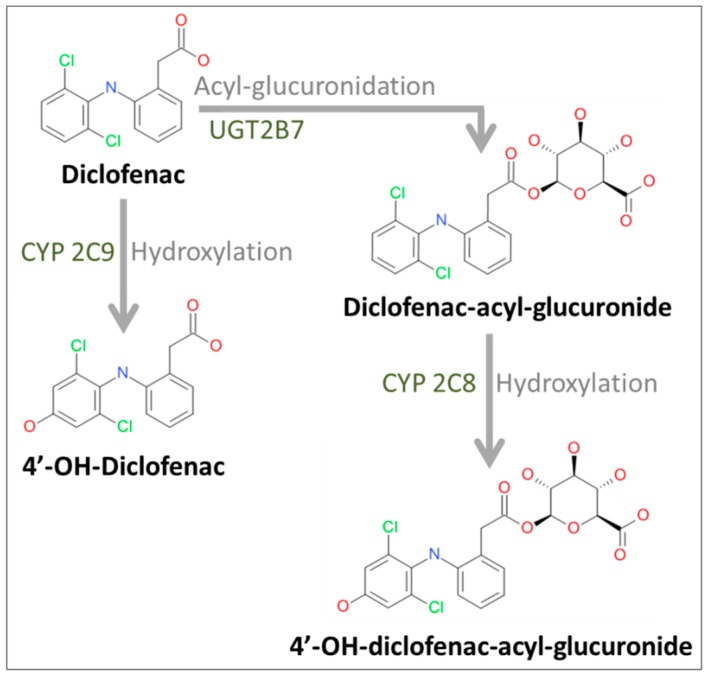
Diclofenac pathways under investigation. The parent drug diclofenac can be hydroxylated by cytochrome P450 (CYP) isoform CYP2C9 to form 4′-OH-diclofenac or glucuronidated by uridine diphosphate (UDP)-glucuronosyltransferase-2B7 (UGT2B7) to form diclofenac-acyl-glucuronide, which can be further hydroxylated by CYP2C8 to 4′-OH-diclofenac-acyl-glucuronide.

In this study, the metabolism and toxicity of the non-steroidal anti-inflammatory drug diclofenac were investigated. In humans, diclofenac is mainly hydroxylated by cytochrome P450 2C9 (CYP2C9) to form the oxidative metabolite 4′-OH-diclofenac [16]. In addition, diclofenac undergoes glucuronidation by uridine diphosphate (UDP)-glucuronosyltransferase-2B7 (UGT2B7) to form the unstable diclofenac-acyl-glucuronide, which can be further hydroxylated by CYP2C8 leading to 4′-OH-diclofenac-acyl-glucuronide [17] (Figure 2).

Diclofenac can cause idiosyncratic drug-induced liver injury (DILI), which is barely predictable in terms of manifestation and dose-dependency of effects [18]. In a study with 180 patients, diclofenac hepatotoxicity was observed in 85% of cases within six months of therapy [19]. Liver injury was characterized by clinical symptoms (e.g., jaundice) and/or changes in biochemical values (e.g., increased transaminase values) [19]. The mechanisms of diclofenac toxicity are not yet completely understood [20], although several hazards caused by its reactive adducts were identified including oxidative stress [17], mitochondrial toxicity [20,21] as well as induction of an immune response via hapten formation [22].

**Figure 3:**
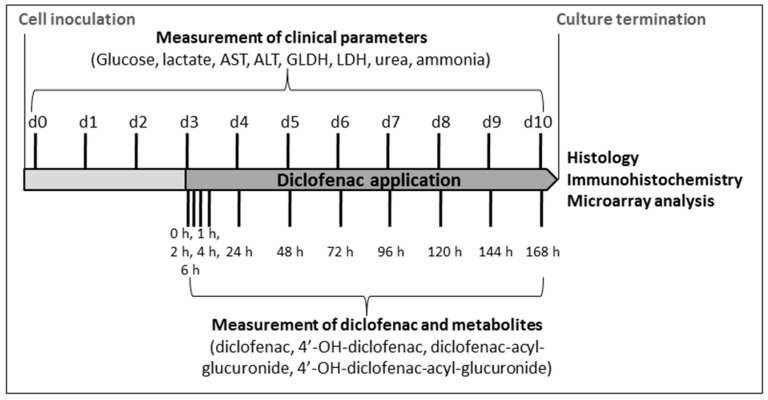
Experimental design. Overview of the time schedule of drug application and sampling in bioreactors run with primary human liver cells.

Hence, diclofenac was selected as a model drug to investigate the suitability of the bioreactor technology for studies on hepatotoxicity. Two concentrations of diclofenac were used to identify a potential dose-dependent toxic effect of the model drug with respect to the parameters determined. The concentrations were chosen on the basis of studies by other groups performed in 2D or 3D culture systems. Several studies showed a toxic effect of diclofenac in the range of 180–1000 µM upon 24 h of exposure based on viability parameters such as 3-(4,5-dimethylthiazol-2-yl)-2,5-diphenyltetrazolium bromide (MTT) assay or lactate dehydrogenase (LDH) release [23,24,25]. Thus, one dose at the lower end (300 µM bioreactors (300 µM BR)) and one dose at the higher end (1000 µM BR) of that range were selected. Diclofenac was applied to primary human liver cells cultured in the bioreactors, and a possible toxic effect of drug exposure was assessed in comparison to untreated control bioreactors (control BR). This was performed by monitoring the metabolic activity of the cells over time with regard to glucose and lactate metabolism and nitrogen elimination (urea and ammonia production). In addition, the leakage of enzymes including alanine transaminase (ALT), aspartate transaminase (AST), LDH and glutamate dehydrogenase (GLDH) was measured to detect potential cell damage caused by the drug. The influence of diclofenac exposure on specific metabolism of the drug was analyzed by measuring the formation of diclofenac products. Upon culture termination the reorganization and differentiation state of the liver cells within the bioreactor was evaluated by means of histological and immunohistochemical analysis. In order to identify potential effects of diclofenac application on metabolic pathways and involved enzymes, the expression of genes assumed to be relevant in the disposition and/or hepatotoxicity of diclofenac were analyzed by whole genome microarray analysis. The design and time schedule of experiments is shown in Figure 3.

## 2. Results

### 2.1. General Metabolic Activity

**Figure 4:**
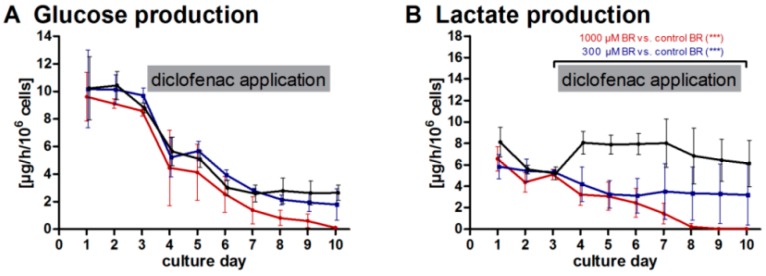

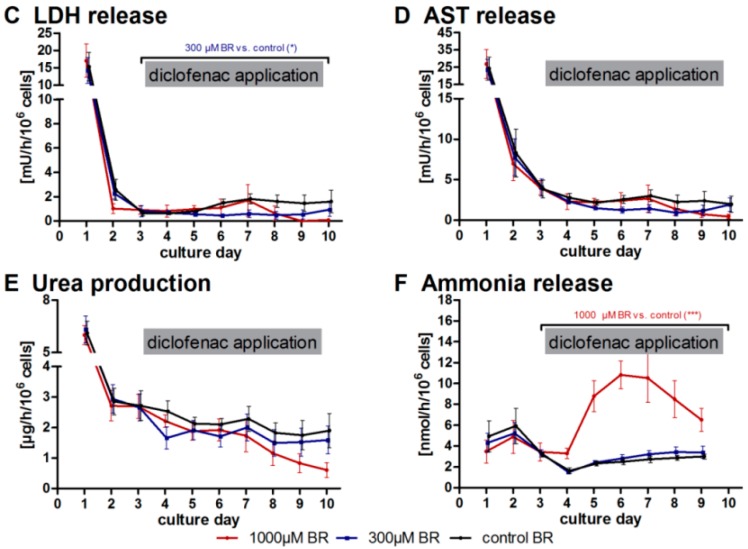
Time-course of clinical parameters in bioreactors with primary human liver cells treated or non-treated with diclofenac. Release respectively production rates in 300 µM bioreactors (300 µM BR) (**blue**) or 1000 µM bioreactors (1000 µM BR) (**red**) or in the non-treated control bioreactors (control BR) (**black**) were determined before (from Day 0 until 3) and during diclofenac application (from Day 3 until 10): production rates of glucose (**A**) and lactate (**B**) were measured as indicators for the overall metabolic performance; lactate dehydrogenase (LDH, **C**) and aspartate transaminase (AST, **D**) were determined as indicators of potential cell damage; and urea production (**E**) and ammonia release (**F**) as parameters for nitrogen metabolism. Values were normalized to 10^6^ inoculated cells. The influence of the drug dose (Day 3–10) on the metabolic activity of the cells in comparison to the control was analyzed by means of one-way analysis of variance (ANOVA) with Dunnett’s multiple comparison test and significant changes (* = *p* < 0.05, *** = *p* < 0.001) are indicated in the graphs (*n* = 3, mean ± SEM).

The metabolic activities of liver cells in bioreactors exposed or non-exposed to diclofenac were monitored throughout the entire cultivation period (Figure 4). After the initial adaptation phase (Day 0–3), the glucose production rate (Figure 4A) decreased in all groups from Day 3 until the end of culture. While the bioreactors exposed to 300 µM (300 µM BR) showed a comparable time-course than the control group (control BR), bioreactors exposed to 1000 µM diclofenac (1000 µM BR) showed a more distinct, though not significantly different, decrease in glucose production starting from Day 4. The influence of drug application was more pronounced regarding lactate production rates (Figure 4B), which showed a significant (*p* < 0.001) dose-dependent decrease in bioreactors exposed to diclofenac as compared to the untreated control group. Release rates of the enzymes LDH and AST (Figure 4C,D) as well as GLDH and ALT (Figure S1) showed similar kinetics in all groups until Day 7 with initial peaks followed by a decrease to basal levels. Only LDH release was significantly lower in the 300 µM BR as compared to the control BR (*p* < 0.05). From Day 7 on, however, values showed a stronger decrease in the 1000 µM BR than in the other groups. Similarly, the production of urea (Figure 4E) showed a distinct, although not significant, decrease from Day 7 in the 1000 µM BR, while both the control BR and the 300 µM BR preserved stable values. The measured ammonia secretion rate showed an initial peak in all groups, and returned to basal values on Day 4 (Figure 4F). At later time-points, both bioreactor groups exposed to the drug differed significantly from the control runs. While the 300 µM BR showed a similar course of ammonia release as the control BR, a drastic increase in ammonia concentration was observed in the 1000 µM BR (*p* < 0.001).

### 2.2. Formation of Diclofenac Metabolites

**Figure 5:**
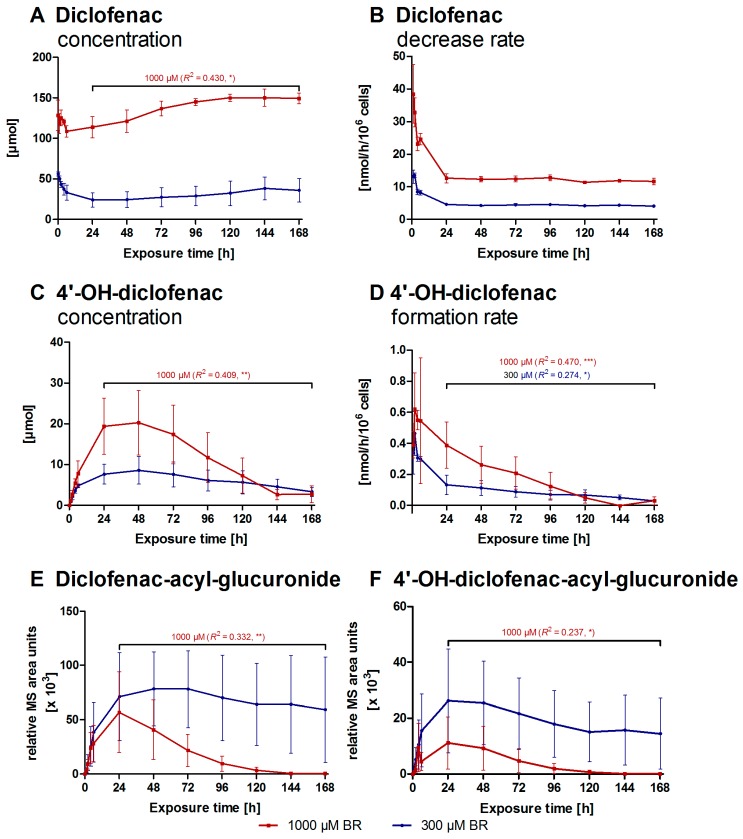
Conversion of diclofenac to specific metabolites in bioreactors with primary human liver cells: diclofenac concentrations (**A**); diclofenac decrease rates (**B**); 4′-OH-diclofenac concentrations (**C**); and 4′-OH-diclofenac formation rates (**D**) as well as mass spectrometry (MS) area units of diclofenac-acyl-glucuronide (**E**) and 4′-OH-diclofenac-acyl-glucuronide (**F**) were determined in 300 µM bioreactors (300 µM BR) (**blue**) or 1000 µM bioreactors (1000 µM BR) (**red**) from Day 3 to 10 of culture. Values were normalized to 10^6^ inoculated cells. The course over time (Day 3–10) of diclofenac metabolism was analyzed by linear regression analysis and significant changes (* = *p* < 0.05, ** = *p* < 0.01, *** = *p* < 0.001) as well as respective regression coefficients are indicated in the graphs. (*n* = 3, mean ± SEM).

Samples for determining the parent drug diclofenac and its metabolites were taken at defined time-points from drug-treated bioreactors in accordance to the sample schedule (see Figure 3). As shown in Figure 5A, an initial diclofenac concentration of 56.3 ± 2.5 µM in the 300 µM BR respectively 128.4 ± 18.7 µM in the 1000 µM BR was measured directly after application to the system (Day 3, 0 h), *i.e.*, in average only 16% of the desired concentration was achieved.

During the first six hours of exposure the concentration of diclofenac decreased rapidly in both groups (Figure 5A). In the 300 µM BR a steady state was maintained between 24 and 96 h of exposure followed by a slow increase. In contrast, a significant increase with a linear regression of *R*^2^ = 0.430 (*p* < 0.05) was observed for the 1000 µM BR. The drug was rapidly eliminated in both groups within the first four hours with an initial elimination rate of 11.7 ± 1.2 (300 µM BR) or 31.5 ± 5.2 nmol/h/10^6^ cells (1000 µM BR), followed by a stable elimination rate of 4.35 ± 0.37 nmol/h/10^6^ cells when applying the lower dose or 12.2 ± 0.8 nmol/h/10^6^ cells in the 1000 µM BR from 24 h to the end of drug exposure on Day 10 (Figure 5B). All investigated diclofenac metabolites could be detected. The concentration of 4′-OH-diclofenac (Figure 5C) was increased initially in all bioreactors with maximum values of 8.56 ± 3.39 µM for the 300 µM BR and 20.3 ± 8.0 µM for the higher dose (1000 µM BR) after 48 h, which was followed by an significant decrease in the concentration in the 1000 µM BR (*R*^2^ = 0.409, *p* < 0.01). As shown in Figure 5D, the formation rate of 4′-OH-diclofenac was lower in the 300 µM BR with a maximum formation rate of 0.46 ± 0.14 nmol/h/10^6^ cells after two hours as compared to the 1000 µM BR with 0.62 ± 0.23 nmol/h/10^6^ cells. Afterwards, a significant decrease was observed in both groups, (300 µM BR: *R*^2^ = 0.274, *p* < 0.05; 1000 µM BR: *R*^2^ = 0.470, *p* < 0.001) reaching similar values after 120 h. Due to the absence of available metabolite reference standards, the formation of diclofenac-acyl-glucuronide (Figure 5E) was determined by mass spectrometry (MS) area units. These showed an increase to a maximum of 78.4 ± 34.1 × 10^3^ relative MS area units at 48 h in the 300 µM BR and maintained a stable level of approximately 67.1 ± 41.2 × 10^3^ relative MS area units afterwards. In contrast, the maximal formation of this metabolite in the higher dose bioreactors was already measured at 24 h after starting with diclofenac exposure (56.9 ± 37.1 × 10^3^ relative MS area units). From Day 4 (24 h), a steep, significant decrease towards nearly zero on Day 8 (120 h) could be detected in the 1000 µM BR (*R*^2^ = 0.332, *p* < 0.01). The formation of 4′-OH-diclofenac-acyl-glucuronide (Figure 5F) showed a comparable time-course in both groups with a maximum amount of 26.2 ± 18.6 × 10^3^ relative MS area units for the 300 µM BR respectively 11.2 ± 9.3 × 10^3^ relative MS area units for the 1000 µM BR at Day 4 (24 h) followed by a decrease, which showed statistical significance only in the 1000 µM BR (*R*^2^ = 0.237, *p* < 0.05).

### 2.3. Cell Morphology and Reorganization in the Bioreactor

**Figure 6:**
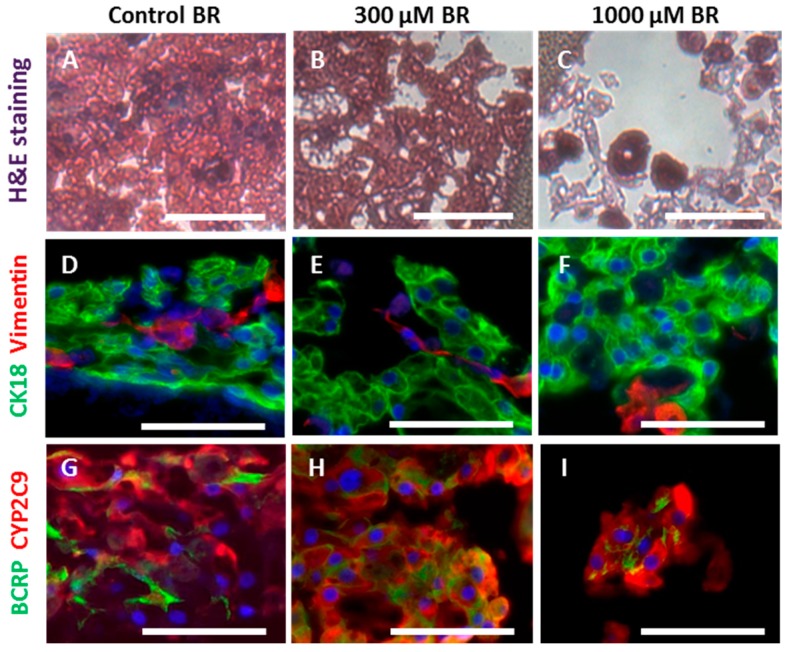
Histological and immunohistochemical analysis of primary human liver cells cultured in bioreactors with or without diclofenac application: cell material from untreated control bioreactors (control BR) (**A**,**D**,**G**); 300 µM bioreactors (300 µM BR) (**B**,**E**,**H**); or 1000 µM bioreactors (1000 µM BR) (**C**,**F**,**I**) was investigated at the end of the experimental phase on Day 10 of culture. The upper row (**A**–**C**) shows hematoxylin and eosin staining of cultures; the middle row (**D**–**F**) shows CK18 (**green**) and vimentin (**red**); and the lower row (**G**–**I**) shows BCRP (**green**) and CYP2C9 (**red**) immunoreactivity. Counterstaining of nuclei is shown in blue (scale bar = 50 µM).

The morphologic appearance and reorganization state of hepatocytes and non-parenchymal liver cells within the bioreactor was investigated after 10 days of cultivation with or without diclofenac exposure by means of histological and immunohistochemical analysis (Figure 6).

The hematoxylin and eosin staining (Figure 6A–C) demonstrated a compact network of cell aggregates within the control BR group (Figure 6A), while diclofenac exposure resulted in partial disruption of aggregates, especially in the 1000 µM BR, which showed a high amount of disintegrated cells (Figure 6C). Hepatocytes characterized by the liver epithelial marker cytokeratin 18 (CK18) and non-parenchymal cells showing vimentin immunoreactivity could be observed in all cultures (Figure 6D–F). The non-parenchymal cells were integrated in the parenchymal aggregates and formed strand-like cell linings in both the control BR and the 300 µM BR (Figure 6D,E), while in the 1000 µM BR the cells appeared less organized. All cultures showed positive staining of the diclofenac metabolizing CYP isoform CYP2C9 located in the cytoplasm of the hepatocytes (Figure 6G–I). In addition, the biliary transporter breast cancer resistance protein (BCRP) was clearly visible in plasma membranes of the pHH (Figure 6G–I). Although all markers under investigation could be detected in all bioreactors, the cell density and the reorganization state of the cells were lower in the 1000 µM BR group.

### 2.4. Analysis of Transcriptomic Data upon Drug Application

**Figure 7:**
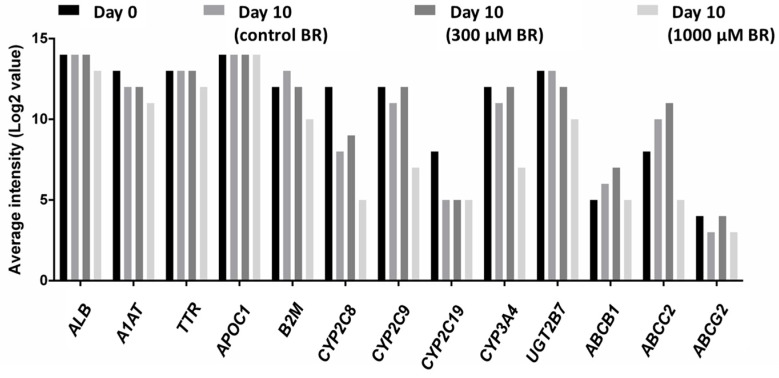
Microarray analysis of primary human liver cells cultured in bioreactors with or without diclofenac exposure. The graph shows average intensities of key genes involved in diclofenac metabolism and diclofenac-mediated toxicity determined in freshly isolated liver cells (Day 0) or cells cultured in bioreactors until Day 10 without diclofenac (control bioreactors (control BR)) or with diclofenac (300 µM bioreactors (300 µM BR)) or 1000 µM bioreactors (1000 µM BR)). Measured genes include albumin (*ALB*), α-1-antitrypsin (*A1AT*), transthyretin (*TTR*), apolipoprotein C-I (*APOC1*), β-2-microglobulin (*B2M*), selected cytochrome P450 (CYP) isoenzymes, uridine diphosphate (UDP) glucuronosyltransferase 2B7 (*UGT2B7*), ATP-binding cassette B1 (*ABCB1*), ATP-binding cassette C2 (*ABCC2*), and ATP-binding cassette G2 (*ABCG2*) (control BR (*n* = 6), 300 µM BR (*n* = 4), 1000 µM BR (*n* = 3)).

At the end of the experiment, total RNA was isolated and whole genome microarray analysis was performed to study specific gene expression profiles. Initial modeling was performed using the donor and dose group as fixed factors to test for possible donor effects. Only genes with an adjusted *p*-value < 0.05 were considered significant. This analysis highlighted only a modest number of genes, which differed between donors across the tested dose range, *i.e.*, 11 genes or 0.06% of analyzed genes (Table S1). Relative average expression levels of selected basal hepatocyte function markers, and relevant drug metabolizing enzymes and transporters involved in the disposition and excretion of diclofenac are shown in Figure 7. The hepatic phenotype of primary human liver cells, assessed by expression levels of liver-specific marker genes over time, was well maintained in the system for the entire duration of the experiment and displayed good correlation with the expression levels in freshly isolated hepatocytes. Similarly, expression levels of drug metabolizing enzymes known to be important in the metabolism of diclofenac (*CYP2C9*, *CYP3A4*, *UGT2B7*, ATP-binding cassette B1 (*ABCB1*); ATP-binding cassette C2 (*ABCC2*), and ATP-binding cassette G2 (*ABCG2*)) showed a high and stable expression for the entire duration of the experiment, although a pronounced decrease was observed for *CYP2C8* and *CYP2C19* from Day 0 to 10 after cell inoculation (Figure 7).

**Figure 8:**
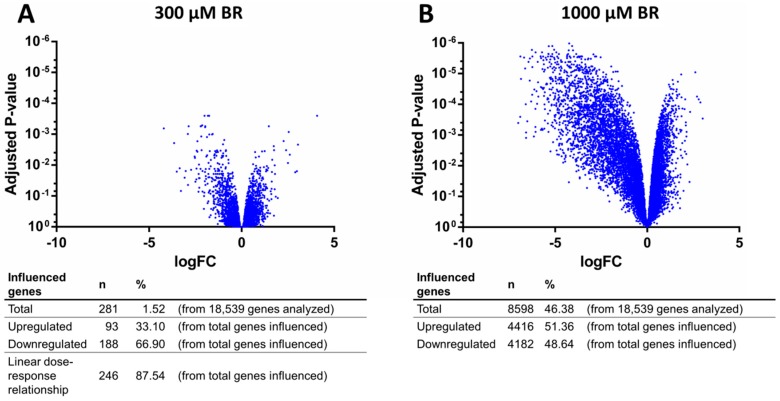
Volcano plot representing the differential expression of all measured genes after exposure to diclofenac: the transcriptional effects of treatment in 300 µM bioreactors (300 µM BR) (**A**) or 1000 µM bioreactors (1000 µM BR) (**B**). The *x*- and *y*-axes represent the magnitude and the significance, respectively, of the transcriptional effect per gene (control bioreactors (control BR) (*n* = 4), 300 µM BR (*n* = 4), 1000 *µ*M BR (*n* = 3), LogFC: Log_2_ fold change in expression as compared to control BR).

Based on the limited inter-donor effects on overall transcription levels, a simplified model was applied to test the effect of diclofenac exposure by using the dose group as a single fixed factor to identify genes differentially affected between the samples tested at a specific dose compared to the control BR. Exposure of the liver cells to diclofenac at concentrations of 300 and 1000 µM resulted in gene expression changes in 281 genes (*i.e*., 1.52%), and 8598 genes (46.38%), respectively, as compared with the untreated control BR (Figure 8A,B). Whereas transcription in the 300 µM BR was primarily downregulated (188 out of 281 genes downregulated—*i.e.*, 66.90%), in the 1000 µM BR approximately equivalent numbers of upregulated (4416 out of 8598 genes—*i.e.*, 51.36%) and downregulated genes (4182 out of 8598 genes—*i.e.*, 48.64%) were found. Nevertheless, transcriptional downregulation was significantly more pronounced, as evidenced by the strong relative-fold downregulation of the 50 most significantly affected genes in the 1000 µM BR group.

Of note, 246 out of 281 genes that differed significantly between the 300 µM BR and the control BR displayed a linear dose-response relationship resulting in an equivalent or greater relative fold change in expression in the 1000 µM BR (Figure 8A), underscoring the possibility of shared regulation pathways. An overview of the most significantly affected genes in the 300 µM BR and the 1000 µM BR is shown in Tables S2 and S3, respectively. To further study the tentative biological effects of diclofenac-induced transcriptional changes, a pathway analysis was performed based on the Gene Ontology (GO) database. In the 300 µM BR group (Table 1), transcriptional changes induced by diclofenac are mainly linked to various immune processes, including chemokine-related chemotaxis (GO:0002687), chemokine receptor binding (GO:0042379), protein activation cascades (GO:0072376) and regulation of leukocyte migration (GO:0002687). Contrastingly, in the 1000 µM BR (Table 2), the strongest effects on gene transcription were observed in pathways related to basal cell functions including regulation of cellular amino acid metabolic processes (GO:0006521), G1 DNA damage checkpoint proteins (GO:0044783), proteasome complex proteins (GO:0000502), oxidoreductase complex proteins (GO:1990204), and respiratory chain proteins (GO:0070469).

With regard to decreased urea production in the 1000 µM BR, a strong negative regulation for ornithine transcarbamoylase (*OTC*), mitochondrial carbamoyl-phosphate synthase 1 (*CPS1*), arginase 1 (*ARG1*), and argininosuccinate lyase (*ASL*) could be detected in the 1000 µM BR. Further, significant effects on the expression of cellular sensors of chemically induced oxidative and electrophilic stress nuclear factor (erythroid-derived 2)-like 2/kelch-like ECH-associated protein 1 (*NRF2/KEAP1*) as well as activating transcription factor 3 (*ATF3*) and growth differentiation factor 15 (*GDF15*) was observed in the 1000 µM BR, whereas these genes were not affected in bioreactors exposed to the lower dose of diclofenac. A complete overview of the magnitude of transcriptional changes expressed as the relative fold-changes in gene expression of individual genes is provided in the Table S4.

## 3. Discussion

Xenobiotics, including drugs, are typically biotransformed in hepatocytes to enhance their renal or biliary excretion. However, the parental drug itself and the formed potential toxic metabolites (*i.e.*, biotoxification) can both lead to hepatotoxicity. Primary human liver cells cultured in a dynamic 3D bioreactor system could be useful to investigate hepatic drug effects *in vitro* with high predictivity. The data from untreated bioreactors (control BR) indicate a stable performance of the hepatocytes subsequent to an adaption phase of three days. This is in consistence with previous studies showing prolonged preservation of hepatocyte functionality in the 3D bioreactor as compared to 2D cultures [14]. A beneficial effect of co-cultivating pHH and non-parenchymal cells (Kupffer and endothelial cells) was also shown in a 3D human liver tissue model [26], emphasizing the importance of the microarchitecture of the liver to maintain cell viability.

To demonstrate the suitability of the bioreactor system for hepatotoxicity studies, diclofenac was selected as a model drug. The drug was applied at two different doses (300 or 1000 µM) over seven days to the system. Previous toxicity assays performed in conventional 2D cultures showed that these concentrations caused a rapid cell death [23,24]. In line with these previous reports, the present study shows a dose-dependent toxic effect of diclofenac based on combined metabolic data, immunohistochemical and gene expression analysis.

Glucose production and lactate production rates determined in the cultures as parameters of energy metabolism showed distinct differences in the cell response to diclofenac application. Whereas the time-course of glucose production rates over the culture time was similar in all bioreactors, with slightly lower values in the 1000 µM BR, lactate production rates showed a clear dose-dependent influence of drug exposure. In contrast to the control BR, which showed stable lactate production rates throughout the culture period, a significant decrease after diclofenac infusion was detected in both drug-treated groups, with reduction of lactate production by approx. 50% in the 300 µM BR, and nearly complete loss of lactate production in the 1000 µM BR. This might be caused by the alterations of mitochondrial functions induced by diclofenac [27]. The value of these basal metabolism parameters was previously confirmed by Prill *et al.* [28], who demonstrated the suitability of continuous glucose and lactate monitoring as an evaluation parameter for high-throughput microbioreactors. An additional option for measuring metabolic activities of the cells in the bioreactor can be seen in resazurin-based cell viability assays, like CellTiter-Blue^®^ or alamarBlue^®^. It was previously shown that the resazurin assay can be repeatedly applied to bioreactor cultures with HepG2 or pHH [29]. However, other studies showed a toxic effect of resazurin upon long-term exposure [30,31], which could impair the effect of diclofenac exposure.

Further, a panel of liver enzymes (AST, ALT, GLDH and LDH) was measured. These enzymes are released upon membrane leakage in case of cell damage and their elevation in plasma is characteristic for liver injury [32,33]. In this study no distinct effects on liver enzyme release of drug application was observed. After a high initial release the rates decreased and reached stable values on a basal level from Day 5 onwards. This is in consistence with results from Wang *et al.* [34], who found an absence of enzyme release in rat hepatocytes treated with diclofenac in 2D cultures. The high release rates during the initial culture phase can most likely be attributed to cell stress as a consequence resulting from cell isolation. Liberation of large amounts of enzymes can result in an exhaustion of enzyme stores preventing further increase upon drug application. Only LDH release was significantly different between the 300 µM BR and the control BR, with lower levels for the 300 µM BR. This can be interpreted as a sign of a stabilizing metabolism with production of LDH under control conditions.

Active nitrogen elimination by the hepatocytes via deamination is indicated by stable urea production rates in the control BR and in the 300 µM BR. In contrast, urea production decreased over time in the 1000 µM BR demonstrating the harmful effect on cell metabolism of higher concentrations of diclofenac. This is in accordance with findings of Borlak *et al.* [35], who recently suggested urea as a robust marker to predict hepatocellular stress during *in vitro* preclinical studies. The disturbed detoxification of nitrogen is confirmed by the significant increase of the ammonia release observed in the 1000 µM BR with values elevated by five-fold compared to the control BR. The accumulation of the toxic base ammonia can further contribute to cell death upon toxic drug exposure. Thus, the results from biochemical parameters indicate that lactate production rate as a marker for energy metabolism and ammonia release in combination with the urea production rate as markers for nitrogen metabolism provide useful markers for evaluation of the cell response to toxic drug exposure.

Single application of diclofenac at a therapeutic dose of 75 mg leads to a maximum plasma concentration of 6.4 µM diclofenac [36]. In clinical reports hepatotoxicity of diclofenac was observed in some patients at a daily dose in the range of 75–200 mg applied over weeks to months [37,38,39]*. In vitro* concentrations tested with respect to hepatotoxicity range between 10 and 1000 µM, with a toxic effect of diclofenac being detected at concentrations between 180 and 1000 µM upon 24 h of exposure [23,24,25,26]. Messner *et al.* [26] reported an IC_50_ value of 178.6 µM after diclofenac treatment of liver microtissues over 14 days, while Bort *et al.* [23] and Lauer *et al.* [25] observed higher values, with an IC_50_ of 331 ± 7 µM and a TC_50_ of 222 ± 142 µM, respectively, which is in the range of the dose of 300 µM used in the present study. However, the direct comparison of data from different studies is difficult due to differences in the culture system, culture condition and assays applied for assessment of toxicity. In addition, potential adsorption processes to culture materials may influence the free concentration of drugs applied. This assumption is supported by the fact that the free diclofenac levels measured directly after application to the bioreactor system (Day 3, 0 h) amounted only to 15% of the expected concentration. Thus, the results indicate a significant loss of the substance in the system, probably due to drug adsorption to the capillary membranes of the bioreactor and/or binding to membrane-bound and soluble proteins [17].

After an initial decrease of the free diclofenac concentration in both bioreactors the free diclofenac concentration increased rapidly in the 1000 µM BR towards the starting concentration. In contrast, nearly constant conversion of diclofenac was observed in the 300 µM BR until the end of culture, amounting to around 60% of the initial drug concentration. The finding that the observed increase is more pronounced in the 1000 µM BR than in the 300 µM BR indicates an impaired cell metabolism in association with a decreased conversion of diclofenac, in addition to potential adsorption processes. While all metabolites under investigation could be detected in the culture perfusates, the time-course of metabolites showed a different behavior in 300 or 1000 µM BR. Time-courses of 4′-OH-diclofenac indicate that pHH in bioreactors actively metabolized diclofenac via CYP2C9 dependent oxidation, since a clear dose-dependent formation rate was detected with much higher values in the 1000 µM BR. This finding is consistent with observed metabolite profiles of excretory pathways in humans, indicating that the *in vivo* clearance in humans is with around 50% mainly based on the oxidative metabolism [16]. The primary metabolite 4′-OH-diclofenac can be further oxidized resulting in an electrophilic quinone imine, which may influence the redox cycling of the cells, produce oxidative stress and can covalently bind to various molecules because of its thiol reactivity [17]. As a consequence, disruption of the balance of cell metabolism can occur. Thus, the continuous decrease of 4′-OH-diclofenac formation observed in the 1000 µM BR may partly be caused by toxic effects of the secondary quinone imine product. Since covalent protein binding was not assessed in the current study, it cannot be confirmed that this proposed mechanism was indeed a contributing factor to the observed toxic effects of diclofenac in the bioreactor system. The formation of diclofenac-acyl-glucuronides and 4′-OH-diclofenac-acyl-glucuronides catalyzed by UGT2B7 and CYP2C8, respectively, showed a similar relatively stable time-course at the lower dose application, and a distinct decrease in the 1000 µM BR, confirming the toxic effect of high concentrations of diclofenac and its metabolites on the activities of drug metabolizing enzymes. Due to their labile ester bond acyl-glucuronides are capable of covalent binding to proteins, reducing their function [40] or even result in immune-mediated destruction of hepatocytes [41]. The high standard deviations especially in the results of diclofenac biotransformation could be potentially resulting from inter-donor variances in enzyme activities, e.g., CYP2C9 activity [42]. While such variances make the statistical evaluation of data from pHH difficult, they also provide the option to reproduce the range of functions and enzyme expression in different patients [2]. In contrast, hepatic cell lines like HepG2 display a reduced expression of CYP enzymes [43], which may impair the hepatotoxic response as previously shown by Rodrigues *et al.* [44].

Histological and immunohistochemical studies performed after 10 days of cultivation showed tissue-like aggregates of pHH with strand-like cell linings of non-parenchymal cells, confirming supportive conditions for liver cell reorganization in the bioreactor system. In addition, CYP2C9, which is mainly responsible for diclofenac metabolism [17], and also the transporter of the parent drug, namely BCRP [45], were well-preserved in bioreactors after cultivation. This physiological *in vitro* representation of *in vivo* liver tissue with a heterogeneous cell population made of parenchymal and non-parenchymal cells is important for clinical-relevant hepatotoxicity testing. It has been shown that the activation of inflammatory cells can enhance DILI [46]. In another study, a protective effect of cell populations like Kupffer cells against drug exposure was observed [47].

Stable preservation of hepatic markers in the bioreactor system was confirmed by microarray analyses showing that the hepatic phenotype of donor cells, assessed by expression levels of liver-specific marker genes (including albumin, α-1-antitrypsin, transthyretin, apolipoprotein C-I and β-2-microglobulin), was well-maintained in the system for the entire duration of the experiment and correlated to the expression levels in freshly isolated liver cells, in accordance with previous studies [14,15]. This is a major advantage over conventional 2D cultivation where a rapid and marked decrease of gene expression encoding for CYP was observed as compared to those expressed in freshly isolated human hepatocytes [4]. To further identify differential effects of diclofenac dose on overall cell function in the bioreactor system, a transcriptomics analysis was performed. Although distinct differences could be detected between control bioreactors and bioreactors exposed to increasing doses of diclofenac, it should be noted, however, that the current study may lack a bona fide time-dose response relation to allow lege artis delineation of potential mechanistic pathways underlying diclofenac toxicity in hepatocytes, and thus these data should be interpreted with caution. After seven days of continuous exposure to diclofenac, cells in the 300 µM BR displayed transcriptional changes in various immune processes, emphasizing the possible role of immune-mediated toxicity in the development of diclofenac induced liver injury [17]. In contrast, in the 1000 µM BR a broad range of basal cell function pathways displayed strong dysregulation (including G1 DNA damage checkpoint proteins, mitochondrial respiratory chain enzymes, and proteasome function), which likely reflects the overall toxic insult of long term exposure. Although these observed changes may likely not be relevant readouts for the initiating effects of diclofenac during initial exposure, the strong observed dose-dependent changes in the canonical toxicity pathways associated with diclofenac do indeed support a faithful representation of previously published pathways involved in the perfused 3D bioreactors [48].

These observations are also corroborated by Zhang *et al.* [49] who recently reported the use of the TG-GATES database to identify a set of four marker genes (early growth response protein 1 (*EGR1*), *ATF3*, *GDF15* and fibroblast growth factor 21 (*FGF21*)) with the potential to serve as biomarkers for prediction of liver toxicity. In line with Zhang *et al.* [48,50], a pronounced transcriptional effect for *ATF3* and *GDF15* was observed in the 1000 µM BR, whereas these genes were not changed in the 300 µM group. Similarly, a distinct change in the expression of *NRF2/KEAP1* was detected, which is in line with studies on HepG2 cells showing that oxidative stress and cytokine signaling based on the activation of the transcription factor *NRF2* arise as a result of adverse drug reaction.

## 4. Materials and Methods

### 4.1. Bioreactor Technology

The used multi-compartment bioreactor consists of three independent but interwoven hollow-fiber capillary systems: two counter-currently perfused medium compartments and one gas compartment that serves for decentralized oxygenation (Figure 1). The cells are located in the fourth compartment, the space formed by the capillary network. A detailed description of the technology can be found elsewhere [15]. In modification of the previously described bioreactor model the bioreactor used in this study contains four capillary layers instead of two layers resulting in a cell compartment volume of 0.5 mL. After connection to a sterile tubing system, the bioreactors were operated in a perfusion device enabling the regulation of medium flow rates and temperature. An integrated electronically operated gas-mixing unit was used for the control of air and CO_2_-flow rates. Bioreactors, tubing systems and perfusion devices were manufactured by Stem Cell Systems, Berlin, Germany.

### 4.2. Isolation of Primary Human Liver Cells

Primary human liver cells were isolated from resected liver tissue from partial hepatectomies with written consent of the patients. The study was conducted in accordance with the Declaration of Helsinki, and the protocol was approved by the Ethics Committee of Charité—Universitätsmedizin Berlin (approval dated 11 April 2013; Project identification code: European Union’s Seventh Framework Programme (FP7/2007-2013EU)). Cell isolation was performed using a two-step collagenase perfusion technique [51] with modifications as described by Pfeiffer *et al.* [52]. Three cell preparations from individual donors with a viability between 79% and 84% were included in this study.

### 4.3. 3D Culture of Primary Human Liver Cells

At least 24 h prior to cell inoculation the bioreactors and connected tubing systems (total system volume: 14 mL) were filled with liver cell culture medium (Heparmed Vito143, Biochrom, Berlin, Germany). The culture medium was supplemented with 20 IU/L insulin, 5 mg/L transferrin, 3 mg/L glucagon, 100,000 U/L penicillin, and 100 mg/L streptomycin (all Biochrom) before use. A number of 4 × 10^7^ total cells from each cell preparation were suspended in up to 4 mL culture medium and inoculated into three parallel bioreactors. The perfusion parameters were set to 3 mL/min for the recirculation flow rate, 0.5 mL/h for the fresh medium flow rate and 5 mL/min for the gas flow rate. The pH value was measured on a daily basis or more frequently, if needed, with a clinical analyzer (ABL 700, Radiometer, Copenhagen, Denmark) and kept between 7.35 and 7.45 by varying the amount of CO_2_ in the range of 4% and 5.5%. The bioreactors were maintained at 37 °C over 10 days. On Day 3 of culture, the drug-free culture medium was exchanged with culture medium containing 300 µM (300 µM BR) or 1000 µM (1000 µM BR) diclofenac sodium salt (Sigma-Aldrich, St. Louis, MO, USA). The perfusion with diclofenac-containing medium was continued until the end of the culture period on Day 10. The third bioreactor (control BR), a non-treated control, was flushed as well and run in parallel without continuous diclofenac exposure.

### 4.4. Analysis of Clinical Parameters

For determination of the metabolic activity of the cells, samples from culture perfusates (for measurement of glucose, lactate, urea and enzyme release) or from the medium outlet (for ammonia measurement) were taken daily according to the time schedule shown in Figure 2. Measurements of glucose and lactate concentrations were performed with a blood gas analyzer (ABL 700, Radiometer). Urea and ammonia concentrations, as well as enzyme activities of AST, ALT, LDH and GLDH were determined with an automated clinical chemistry analyzer (Cobas^®^ 8000, Roche Diagnostics GmbH, Mannheim, Germany). The calculation of daily production or release rates was performed on the basis of the total system volume and feed rate as described by Hoffmann *et al.* [14].

### 4.5. Analysis of Diclofenac and Its Metabolites

Following diclofenac application on culture Day 3, samples from the culture perfusates were taken at 0, 1, 2, 4, 6, 24, 48, 72, 96, 120, 144 and 168 h according to the time schedule shown in Figure 3 for quantification of the parent drug (diclofenac) and formed metabolites (4′-OH-diclofenac, diclofenac-acyl-glucuronide, and 4′-OH-diclofenac-acyl-glucuronide) by liquid chromatography-mass spectrometry/mass spectrometry (LC-MS/MS). Medium probes were mixed in a 1:1 ratio (*v*:*v*) either with dimethyl sulfoxide ((DMSO), Carl Roth, Karlsruhe, Germany) for analysis of 4′-OH-diclofenac or with DMSO containing 10% formic acid (Sigma-Aldrich) for analysis of acyl-glucuronide. Subsequently, samples were snap-frozen and stored at −80 °C until further analysis. Samples were injected using acetonitrile and ultrapure water as organic and aqueous mobile phases, respectively. Both mobile phases were fortified with 0.1% (*v*:*v*) formic acid. Separation was carried out using an Acquity UPLC column (BEH C18 (1.7 µm) 50 × 2.1 mm ID, column temperature = 50 °C) and eluted fractions were directly passed through a Xevo TQ-S tandem mass spectrometer (Waters Corp., Milford, MA, USA) operated in negative electrospray ionization mode. Acquired data were processed with Thermo Xcaliber software (Thermo Scientific, Waltham, MA, USA).

### 4.6. Histology and Immunohistochemistry

Upon termination of bioreactor cultures on Day 10 of culture, bioreactors were opened and sections of the capillary bed containing the cell material were taken. Samples were fixed in 4% formalin solution (Herbeta Arzneimittel, Berlin, Germany), dehydrated and embedded in paraffin. Slides of 2.5-µM thickness were deparaffinized and rehydrated. For histological analysis slides were shortly rinsed with distilled water, incubated for 20 min with Harris’ hematoxylin solution (Sigma-Aldrich), rinsed 3 min under running tap water and incubated for 2 min in eosin solution (Carl Roth). Finally slides were dehydrated and fixed. For immunohistochemistry, initial antigen retrieval was performed by incubation with 0.1 M citrate buffer in a pressure cooker for 15 min, followed by incubation of slides with blocking buffer (phosphate buffered saline (PBS; Life Technologies, Carlsbad, CA, USA) with 2% fetal calf serum (PAA, Pasching, Austria) and 2.5% bovine serum albumin (Sigma-Aldrich) for 1 h at room temperature. Subsequently the sections were incubated for 1 h with primary antibodies (1:100 dilution), followed by 1 h incubation with the secondary antibodies (1:1000 dilution). Monoclonal mouse antibodies against CK18 (ab668, Abcam, Cambridge, UK) or BCRP (ab3380, Abcam), and polyclonal rabbit antibodies against vimentin (sc-5565, Santa Cruz, Santa Cruz, CA, USA) or CYP2C9 (PAP091, Nosan, Yokohama, Japan) were used as primary antibodies. As secondary antibodies, fluorochrome-coupled anti-mouse IgG 488 (A11029; Life Technologies) and anti-rabbit IgG 594 (A11037; Life Technologies) were used. Counterstaining of nuclei was performed using bisbenzimide H 33342 trihydrochloride ((Hoechst 33342); Sigma-Aldrich).

### 4.7. Microarray Analysis

RNA was isolated from the freshly isolated human liver cells used for inoculation of the bioreactors on Day 0 and from bioreactor cultures after termination of the cultures on Day 10. The isolation was performed using RLT buffer (Qiagen, Hilden, Germany) with 1% 2-Mercaptoethanol (Sigma-Aldrich) in conjunction with the RNeasy mini kit (Qiagen). Next, cDNA targets were prepared and labeled using the IVT express kit and then hybridized on an Affymetrix^®^ Human Genome U219 array plate in the GeneTitan^®^ instrument (Affymetrix, Santa Clara, CA, USA) according to the manufacturer’s protocol. Gene expression microarray analysis of all time-points was performed using the Bioconductor package version 2.12 (working with R version 3.0.1, [53]). Grouping of the individual probes into gene-specific probe sets was based on Entrez Gene using the metadata package hgu219hsentrezg (cdf version 19.0.0, [54]), assigning probes to 18539 genes. The robust multi-array average algorithm (RMA, [55]) was applied for preprocessing (probewise background correction, normalization and probeset summarization). Differential gene expression at Day 10 of the bioreactor incubations was analyzed using limma [56,57] using donor ID and/or dose groups as fixed factors. Multiple testing correction was estimated using the method of Benjamini and Hochberg (False Discovery Rate (FDR)) [58]. Pathway information was retrieved from the Gene Ontology Consortium [59]. Pathway analysis is based on MLP [60] using the *p*-values of all genes from the differential analysis.

### 4.8. Statistics

Experiments were performed in triplicate and values are shown as means ± standard errors of the mean (SEM). Statistical analyzes were performed using GraphPad Prism 5.0 for Windows (GraphPad Software, San Diego, CA, USA). The influence of the drug dose (Day 3–10) on the metabolic activity of the cells in comparison to the control was analyzed by means of one-way analysis of variance (ANOVA) with Dunnett’s multiple comparison test. The course over time (Day 3–10) of diclofenac metabolism was analyzed by linear regression analysis. Differences were judged as follows: * = *p* < 0.05, ** = *p* < 0.01, *** = *p* < 0.001. For microarray analyses, genes with an adjusted *p* < 0.05 were considered as differentially expressed in all comparisons unless mentioned otherwise.

## 5. Conclusions

In conclusion, transcriptional data in line with functional readouts clearly showed a dose-response effect of diclofenac in the perfused bioreactor system. Thus, the data underscore the suitability of the perfused 3D bioreactor system to perform long-term toxicity studies *in vitro*. In addition, the structure of the bioreactor perfusion circuit allows for various flow modes mimicking the *in vivo* application of a drug. In case of diclofenac a protocol simulating the continuous application of a drug over a longer time period was established, but also toxicity studies with a single dose application are conceivable without supplying fresh medium and introducing a bolus of the drug to the perfusion circuit. In summary, the 3D bioreactor system represents a valuable tool for *in vitro* studies on hepatic drug toxicity in humans.

## Figures and Tables

**Table 1:** Gene Ontology (GO) gene sets most strongly affected upon treatment with a low dose of diclofenac (300 µM bioreactors).

Rank	GO ID	Size	*p*-Value	Description	Parent
**Gene Ontology Biological Processes**					
1	GO:0072376	67 (90)	6.0 × 10^−33^	Protein activation cascade	
2	GO:0006956	47 (69)	1.8 × 10^−31^	Complement activation	1
3	GO:0016064	94 (119)	9.8 × 10^−29^	Immunoglobulin mediated immune response	
4	GO:0002455	43 (67)	1.0 × 10^−27^	Humoral immune response mediated by circulating immunoglobulin	3
5	GO:0019724	97 (122)	4.9 × 10^−27^	B cell mediated immunity	3
6	GO:0002576	83 (87)	1.4 × 10^−26^	Platelet degranulation	
7	GO:0002687	89 (90)	3.5 × 10^−26^	Positive regulation of leukocyte migration	
8	GO:0006958	31 (52)	2.0 × 10^−22^	Complement activation, classical pathway	1, 2, 3, 4, 5
9	GO:0002688	79 (83)	2.7 × 10^−21^	Regulation of leukocyte chemotaxis	
10	GO:0002690	68 (69)	2.8 × 10^−18^	Positive regulation of leukocyte chemotaxis	7, 9
**Gene Ontology Cellular Component**					
1	GO:0032994	37 (40)	8.7 × 10^−25^	Protein-lipid complex	
2	GO:0034358	35 (38)	1.8 × 10^−24^	Plasma lipoprotein particle	1
3	GO:0042613	12 (16)	4.6 × 10^−24^	MHC class II protein complex	
4	GO:0042611	22 (27)	1.6 × 10^−20^	MHC protein complex	3
5	GO:0031983	73 (82)	1.1 × 10^−19^	Vesicle lumen	
6	GO:0060205	72 (81)	2.4 × 10^−19^	Cytoplasmic membrane-bounded vesicle lumen	5
7	GO:0030134	48 (52)	6.7 × 10^−17^	ER to Golgi transport vesicle	
8	GO:0012507	38 (42)	1.6 × 10^−15^	ER to Golgi transport vesicle membrane	7
9	GO:0034364	23 (26)	1.0 × 10^−14^	High-density lipoprotein particle	1, 2
10	GO:0031091	60 (63)	1.3 × 10^−12^	Platelet α granule	
**Gene Ontology Molecular Functions**					
1	GO:0042379	54 (58)	2.8 × 10^−9^	Chemokine receptor binding	
2	GO:0008009	43 (47)	1.3 × 10^−8^	Chemokine activity	1
3	GO:0030170	53 (56)	5.3 × 10^−7^	Pyridoxal phosphate binding	
4	GO:0003823	76 (107)	2.0 × 10^−6^	Antigen binding	
5	GO:0045236	17 (17)	9.7 × 10^−6^	CXCR chemokine receptor binding	1, 2
6	GO:0023026	15 (16)	2.6 × 10^−5^	MHC class II protein complex binding	4
7	GO:0060228	6 (6)	3.1 × 10^−5^	Phosphatidylcholine-sterol O-acyltransferaseactivator activity	
8	GO:0031210	15 (16)	4.7 × 10^−5^	Phosphatidylcholine binding	
9	GO:0019864	8 (12)	1.1 × 10^−4^	IgG binding	
10	GO:0023023	17 (19)	1.6 × 10^−4^	MHC protein complex binding	4, 6

**Table 2:** Gene Ontology (GO) gene sets most strongly affected upon treatment with a high dose of diclofenac (1000 µM bioreactors).

Rank	GO ID	Size	*p*-Value	Description	Parent
**Gene Ontology Biological Processes**					
1	GO:0042590	74 (79)	1.2 × 10^−46^	Antigen processing and presentation of exogenous peptide antigen via MHC class I	
2	GO:0002479	70 (75)	2.1 × 10^−36^	Antigen processing and presentation of exogenous peptide antigen via MHC class I, TAP-dependent	1
3	GO:0006521	59 (62)	1.0 × 10^−31^	Regulation of cellular amino acid metabolic process	
4	GO:0033238	79 (82)	2.9 × 10^−26^	Regulation of cellular amine metabolic process	
5	GO:0002576	83 (87)	8.9 × 10^−19^	Platelet degranulation	
6	GO:0044783	75 (77)	3.9 × 10^−17^	G1 DNA damage check point	
7	GO:0051437	68 (72)	6.7 × 10^−17^	Positive regulation of ubiquitin-protein ligase activity	
8	GO:0051351	86 (94)	1.4 × 10^−16^	Positive regulation of ligase activity	
9	GO:0051436	63 (67)	2.1 × 10^−16^	Negative regulation of ubiquitin-protein ligase activity	
10	GO:0051443	81 (89)	3.0 × 10^−16^	Positive regulation of ubiquitin-protein transferase activity	8
**Gene Ontology Cellular Component**					
1	GO:0000502	63 (66)	3.6 × 10^−25^	Proteasome complex	
2	GO:1990204	78 (83)	2.6 × 10^−18^	Oxidoreductase complex	
3	GO:0043202	72 (73)	2.5 × 10^−16^	Lysosomal lumen	
4	GO:0005775	73 (77)	1.4 × 10^−15^	Vacuolar lumen	3
5	GO:0031091	60 (63)	3.1 × 10^−15^	Platelet α granule	
6	GO:0070469	65 (72)	3.1 × 10^−15^	Respiratory chain	
7	GO:0005746	59 (65)	9.6 × 10^−13^	Mitochondrial respiratory chain	6
8	GO:0022624	22 (24)	3.9 × 10^−12^	Proteasome accessory complex	1
9	GO:0031902	93 (99)	3.1 × 10^−11^	Late endosome membrane	
10	GO:0005839	20 (20)	1.6 × 10^−10^	Proteasome core complex	1
**Gene Ontology Molecular Functions**					
1	GO:0009055	89 (99)	8.2 × 10^−21^	Electron carrier activity	
2	GO:0003743	48 (52)	1.1 × 10^−14^	Translation initiation factor activity	
3	GO:0016651	87 (95)	9.7 × 10^−14^	Oxidoreductase activity, acting on NAD(P)H	
4	GO:0016655	49 (53)	1.6 × 10^−12^	Oxidoreductase activity, acting on NAD(P)H, quinon or similar compound as acceptor	3
5	GO:0008135	75 (83)	2.9 × 10^−11^	Translation factor activity, nucleic acid binding	
6	GO:0004298	21 (21)	4.3 × 10^−8^	Threonine-type endopeptidase activity	
7	GO:0070003	21 (21)	4.3 × 10^−8^	Threonine-type peptidase activity	6
8	GO:0016830	45 (47)	1.1 × 10^−6^	Carbon-carbon lyase activity	
9	GO:0003954	36 (39)	2.4 × 10^−6^	NADH dehydrogenase activity	3, 4
10	GO:0008137	36 (39)	2.4 × 10^−6^	NADH dehydrogenase (ubiquinone) activity	3, 4, 9

## Supplementary Materials

Supplementary materials can be found at http://www.mdpi.com/1422-0067/17/4/584/s1.

## Abbreviations

2D	two-dimensional
3D	three-dimensional
300 µM BR	300 µM bioreactors
1000 µM BR	1000 µM bioreactors
*A1AT*	α-1-antitrypsin
*ABCB1*	ATP-binding cassette B1
*ABCC2*	ATP-binding cassette C2
*ABCG2*	ATP-binding cassette G2
*ALB*	albumin
ALT	alanine transaminase
*ARG1*	arginase 1
*APOC1*	apolipoprotein C-I
*ASL*	argininosuccinate lyase
*AST*	aspartate transaminase
*ATF3*	activating transcription factor 3
*B2M*	β -2-microglobulin
BCRP	breast cancer resistance protein
CK 18	cytokeratin 18
control BR	control bioreactors
*CPS1*	mitochondrial carbamoyl-phosphate synthase 1
CYP	cytochrome P450
DILI	drug-induced liver injury
DMSO	dimethyl sulfoxide
FDR	False Discovery Rate
GLDH	glutamate dehydrogenase
*GDF15*	growth differentiation factor 15
LDH	lactate dehydrogenase
MS	mass spectrometry
*NRF2/KEAP1*	nuclear factor (erythroid-derived 2)-like 2/kelch-like ECH-associated protein 1
*OTC*	ornithine transcarbamoylase
pHH	primary human hepatocytes
SEM	standard errors of the mean
*TTR*	transthyretin
UDP	uridine diphosphate
UGT2B7	UDP glucuronosyltransferase 2B7
