# Supplementary material for: In Vitro Model for Hepatotoxicity Studies Based on Primary Human Hepatocyte Cultivation in a Perfused 3D Bioreactor System
Source: Int J Mol Sci. 2016 Apr 16;17(4):584. doi: 10.3390/ijms17040584 (PMC4849040; doi:10.3390/ijms17040584)
Supplement: Supplementary file 1 [file ijms-17-00584-s001.zip › ijms-122987-Supplementary Materials/ijms-122987-supplementary information 1.pdf]

# Supplementary Materials: I*n Vitro* Model for Hepatotoxicity Studies Based on Primary Human Hepatocyte Cultivation in a Perfused 3D Bioreactor System

**Fanny Knöspel, Frank Jacobs, Nora Freyer, Georg Damm, An De Bondt, Ilse van den Wyngaert, Jan Snoeys, Mario Monshouwer, Marco Richter, Nadja Strahl, Daniel Seehofer and Katrin Zeilinger**

**Table S1.** Overview of the genes differentially expressed between hepatocyte donors in control bioreactors on Day 10 (LogFC: $Log_2$ fold change in expression).

| Entrez-ID | Symbol | Gene Name | logFC | Adjusted *p*-Value |
|---|---|---|---|---|
| 397 | *ARHGDIB* | Rho GDP dissociation inhibitor (GDI) β | 1.35 | 0.046 |
| 1551 | *CYP3A7* | Cytochrome P450, family 3, subfamily A, polypeptide 7 | 3.94 | 0.046 |
| 4072 | *EPCAM* | Epithelial cell adhesion molecule | 2.40 | 0.046 |
| 4495 | *MT1G* | Metallothionein 1G | −4.92 | 0.046 |
| 4496 | *MT1H* | Metallothionein 1H | −5.85 | 0.0076 |
| 30001 | *ERO1L* | ERO1-like (S. cerevisiae) | 1.05 | 0.0092 |
| 51302 | *CYP39A1* | Cytochrome P450, family 39, subfamily A, polypeptide 1 | 1.075 | 0.0060 |
| 57600 | *FNIP2* | Folliculin interacting protein 2 | −0.74 | 0.039 |
| 64097 | *EPB41L4A* | Erythrocyte membrane protein band 4.1 like 4A | 1.73 | 0.021 |
| 79668 | *PARP8* | Poly (ADP-ribose) polymerase family, member 8 | 0.79 | 0.032 |
| 118663 | *BTBD16* | BTB (POZ) domain containing 16 | 1.17 | 0.046 |

**Table S2.** Overview of the genes most significantly affected upon treatment with 300 μM diclofenac (LogFC: $Log_2$ fold change in expression as compared with control BR).

| Entrez-ID | Symbol | Gene Name | logFC | Adjusted *p*-Value |
|---|---|---|---|---|
| | | **Top 10 Downregulated Genes** | | |
| 3108 | *HLA-DMA* | Major histocompatibility complex, class II, DM α | −2.01 | 0.00025 |
| 3240 | *HP* | Haptoglobin | −1.76 | 0.00025 |
| 7805 | *LAPTM5* | Lysosomal protein transmembrane 5 | −1.82 | 0.00025 |
| 11010 | *GLIPR1* | GLI pathogenesis-related 1 | −2.13 | 0.00052 |
| 2207 | *FCER1G* | Fc fragment of IgE, high affinity I, receptor for; gamma polypeptide | −2.22 | 0.00055 |
| 51156 | *SERPINA10* | Serpin peptidase inhibitor, clade A (α-1 antiproteinase, antitrypsin), member 10 | −2.19 | 0.00055 |
| 6659 | *SOX4* | SRY (sex determining region Y)-box 4 | −1.97 | 0.00055 |
| 6696 | *SPP1* | Secreted phospho protein 1 | −2.86 | 0.00055 |
| 713 | *C1QB* | Complement component 1, q subcomponent, B chain | −1.97 | 0.00059 |
| 6347 | *CCL2* | Chemokine (C–C motif) ligand 2 | −4.20 | 0.00065 |
| | | **Top 10 Upregulated Genes** | | |
| 337 | *APOA4* | Apolipoprotein A-IV | 4.075 | 0.00025 |
| 771 | *CA12* | Carbonic anhydrase XII | 1.47 | 0.00055 |
| 1491 | *CTH* | Cystathionine gamma-lyase | 2.55 | 0.00085 |
| 1649 | *DDIT3* | DNA-damage-inducibletranscript 3 | 2.27 | 0.0017 |
| 3934 | *LCN2* | Lipocalin 2 | 3.050 | 0.0022 |
| 2673 | *GFPT1* | Glutamine-fructose-6-phosphate transaminase 1 | 1.74 | 0.0024 |
| 4953 | *ODC1* | Ornithine decarboxylase 1 | 1.76 | 0.0033 |
| 6520 | *SLC3A2* | Solute carrier family 3 (amino acid transporter heavy chain), member 2 | 2.41 | 0.0033 |
| 3376 | *IARS* | Isoleucyl-tRNA synthetase | 1.25 | 0.0035 |
| 84513 | *PPAPDC1B* | Phosphatidic acid phosphatase type 2 domain containing 1B | 1.11 | 0.0036 |

**Table S3.** Overview of the genes most significantly affected upon treatment with 1000 µM diclofenac (LogFC: $Log_2$ fold change in expression as compared with control BR).

| Entrez-ID | Symbol | Gene Name | logFC | Adjusted *p*-Value |
|---|---|---|---|---|
| **Top 10 Downregulated Genes** | | | | |
| 7805 | *LAPTM5* | Lysosomal protein transmembrane 5 | −6.036 | $1.13 \times 10^{-11}$ |
| 1974 | *EIF4A2* | Eukaryotic translation initiation factor 4A2 | −6.028 | $2.09 \times 10^{-10}$ |
| 3015 | *H2AFZ* | H2A histone family, member Z | −5.44 | $2.31 \times 10^{-10}$ |
| 4069 | *LYZ* | Lysozyme | −5.30 | $8.70 \times 10^{-11}$ |
| 5516 | *PPP2CB* | Protein phosphatase 2, catalytic subunit, β isozyme | −5.71 | $2.38 \times 10^{-10}$ |
| 7941 | *PLA2G7* | Phospholipase A2, group VII (platelet-activating factor acetylhydrolase, plasma) | −4.72 | $1.32 \times 10^{-10}$ |
| 11342 | *RNF13* | Ring fingerprotein 13 | −4.89 | $2.75 \times 10^{-10}$ |
| 6166 | *RPL36AL* | Ribosomal protein L36a-like | −5.56 | $4.64 \times 10^{-10}$ |
| 9516 | *LITAF* | Lipopolysaccharide-induced TNF factor | −4.41 | $4.48 \times 10^{-10}$ |
| 5431 | *POLR2B* | Polymerase (RNA) II (DNA directed) polypeptide B, 140 kDa | −4.24 | $5.60 \times 10^{-10}$ |
| **Top 10 Upregulated Genes** | | | | |
| 1178 | *CLC* | Charcot-Leydencrystalgalectin | 2.61 | $9.02 \times 10^{-6}$ |
| 644809 | *C15orf56* | Chromosome 15 open readingframe 56 | 1.85 | $1.07 \times 10^{-5}$ |
| 203076 | *C8orf74* | Chromosome 8 open readingframe 74 | 1.38 | $1.62 \times 10^{-5}$ |
| 3456 | *IFNB1* | Interferon, β 1, fibroblast | 1.66 | $1.83 \times 10^{-5}$ |
| 91544 | *UBXN11* | UBX domainprotein 11 | 1.41 | $2.19 \times 10^{-5}$ |
| 56253 | *CRTAM* | Cytotoxic and regulatory T cell molecule | 1.18 | $2.28 \times 10^{-5}$ |
| 1133 | *CHRM5* | Cholinergicreceptor, muscarinic 5 | 1.224 | $2.67 \times 10^{-5}$ |
| 5319 | *PLA2G1B* | Phospholipase A2, group IB (pancreas) | 1.27 | $3.14 \times 10^{-5}$ |
| 9153 | *SLC28A2* | Solute carrier family 28 (concentrative nucleoside transporter), member 2 | 1.33 | $3.14 \times 10^{-5}$ |
| 84560 | *MT4* | Metallothionein 4 | 1.60 | $3.26 \times 10^{-5}$ |

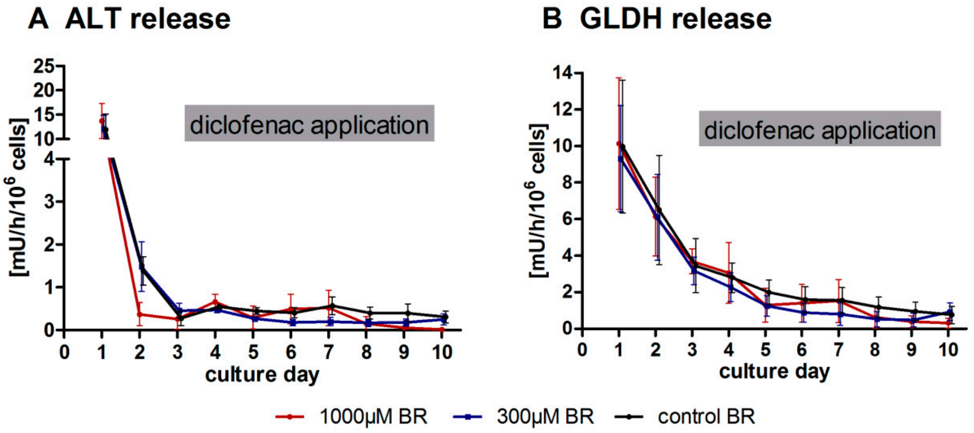


**Figure S1.** Release rates of alanine transaminase (ALT) and glutamate dehydrogenase (GLDH) in bioreactor with primary human liver cells treated or non-treated with diclofenac. Enzyme release rates in 300 µM bioreactors (300 µM BR) (**blue**) or 1000 µM bioreactors (1000 µM BR) (**red**) or in the non-treated control bioreactors (control BR) (**black**) were determined before (from Day 0 to 3) and during diclofenac application (from Day 3 to 10). ALT (**A**) and GLDH (**B**) were measured as indicators of potential cell damage. Values were normalized to $10^6$ inoculated cells. Values were normalized to $10^6$ inoculated cells. The influence of the drug dose (Day 3–10) on the metabolic activity of the cells in comparison to the control was analyzed by means of one-way analysis of variance (ANOVA) with Dunnett's multiple comparison test .($n = 3$, mean ± SEM).
